# Type I Vs. Type II Cytokine Levels as a Function of SOD1 G93A Mouse Amyotrophic Lateral Sclerosis Disease Progression

**DOI:** 10.3389/fncel.2015.00462

**Published:** 2015-12-01

**Authors:** Amilia Jeyachandran, Benjamin Mertens, Eric A. McKissick, Cassie S. Mitchell

**Affiliations:** Department of Biomedical Engineering, Georgia Institute of Technology and Emory UniversityAtlanta, GA, USA

**Keywords:** amyotrophic lateral sclerosis, inflammation, cytokine, interleukin, GFAP, TNF-α, G93A, SOD1

## Abstract

Amyotrophic Lateral Sclerosis (ALS) is a fatal motoneuron disease that is characterized by the degradation of neurons throughout the central nervous system. Inflammation have been cited a key contributor to ALS neurodegeneration, but the timeline of cytokine upregulation remains unresolved. The goal of this study was to temporally examine the correlation between the varying levels of pro-inflammatory type I cytokines (IL-1β, IL-1α, IL-12, TNF-α, and GFAP) and anti-inflammatory type II cytokines (IL-4, IL-6, IL-10) throughout the progression of ALS in the SOD1 G93A mouse model. Cytokine level data from high copy SOD1 G93A transgenic mice was collected from 66 peer-reviewed studies. For each corresponding experimental time point, the ratio of transgenic to wild type (TG/WT) cytokine was calculated. One-way ANOVA and *t*-tests with Bonferonni correction were used to analyze the data. Meta-analysis was performed for four discrete stages: early, pre-onset, post-onset, and end stage. A significant increase in TG cytokine levels was found when compared to WT cytokine levels across the entire SOD1 G93A lifespan for majority of the cytokines. The rates of change of the individual cytokines, and type I and type II were not significantly different; however, the mean fold change of type I was expressed at significantly higher levels than type II levels across all stages with the difference between the means becoming more pronounced at the end stage. An overexpression of cytokines occurred both before and after the onset of ALS symptoms. The trend between pro-inflammatory type I and type II cytokine mean levels indicate a progressive instability of the dynamic balance between pro- and anti-inflammatory cytokines as anti-inflammatory cytokines fail to mediate the pronounced increase in pro-inflammatory cytokines. Very early immunoregulatory treatment is necessary to successfully interrupt ALS-induced neuroinflammation.

## Introduction

Amyotrophic Lateral Sclerosis (ALS) is a fatal neurodegenerative disease that is characterized by the rapid degradation of motor neurons over the course of the disease, resulting in paralysis, respiratory failure, and ultimately death. ALS is multi-faceted pathophysiology, which includes axonal transport deficiency; upregulation of apoptotic cascades; changes in cellular chemistry, including metallation and enzymes; cellular energetics deficiencies; excitability, including changes in neurotransmitters and transporters; inflammation, including increased microglia activation and gliosis; oxidative stress, including increases in free intracellular oxidants and anti-oxidants; protein deregulation, including increased protein aggregates and decreased autophagy; and systemic contributors, including those of muscular and non-neuromuscular origin (Irvin et al., 2015; Kim et al., 2015). For a recent in-depth informatics-based review of the entire SOD1 G93A field, including an overview of the nine previously mentioned pathophysiological categories, please see (Kim et al., 2015). Inflammation, or more specifically neuroinflammation, is one of the more heavily researched ALS contributors (Kim et al., 2015), which is frequently assessed in the familial or transgenic superoxide dismutase-1 guanine 93 to alanine (SOD1 G93A) murine model (Pfohl et al., 2015). Although the crucial role of neuroinflammation in the pathology of ALS is evident, the extent to which the inflammatory response is neurotoxic, and the balance of the inflammatory regulation and deregulation over the course of the disease progression remain unclear (McCombe and Henderson, 2011; Evans et al., 2013).

Currently, the drug Riluzole has shown to be slightly effective in extending patient survival by 75 days through targeting inflammation caused by glutamate (Riviere et al., 1998; McGeer and McGeer, 2002). Other inflammatory treatments have also shown to significantly increase life expectancy. The inflammatory enzyme COX-2 has been found in high levels in G93A mice (Almer et al., 2001), and *in vivo* trials in mice using COX-2 inhibitors have yielded great results, extending life expectancy by upwards of 20% (Drachman et al., 2002). Possible treatments reducing pathological inflammation regulation may be used in conjunction to achieve a greater impact in the treatment of ALS (McGeer and McGeer, 2002).

Cytokines, the primary messenger molecules of the inflammatory response, are released from leukocytes, microglia, and astrocytes (Hart, 2003). In a non-pathological state, cytokines use complex signaling cascades in order to yield a balanced and non-toxic, protective immune response to the target site (Greenhalgh and Hilton, 2001). Type I cytokines increase the inflammatory response (i.e., type I = pro-inflammatory), while type II cytokines decrease the inflammatory response (i.e., type II = anti-inflammatory; Dong et al., 2011). However, pathological overexpression of cytokines or disturbance of their intricate balance can cause the overall inflammatory response to be damaging rather than protective (Papadimitriou et al., 2010). Possible primary inducers of ALS inflammation include glutamate excitotoxicity and oxidative stress due to free radical accumulation (Mitchell and Lee, 2012; Kim et al., 2015). Primary and secondary activation of microglia and astrocytes further exacerbates the release of pro-inflammatory cytokines and nitric oxide through a positive feedback loop (Mitchell and Lee, 2008).

Upregulation of pro-inflammatory cytokines is thought to be associated with an increased severity of symptoms in ALS (Nguyen et al., 2004). However, the exact timeline of cytokine disturbance remains unclear (McCombe and Henderson, 2011). In this study, we present a quantitative, comprehensive examination of cytokines over the entire course of the SOD1 G93A transgenic mouse ALS disease progression. Moreover, we assess the relationship between type I and type II cytokines as a function of ALS progression. We hypothesized that type I cytokine levels would increase significantly in comparison to type II cytokine levels at each disease stage with this difference more pronounced in the latter stages of the disease. Our meta-analysis includes data from 66 peer-reviewed experimental studies, which measured cytokine levels in both transgenic G93A SOD1 mice (TG) and in wild type mice (WT). The fold changes, with respect to WT levels, of cytokines are compared across the lifespan of the SOD1 G93A TG mouse, using four discrete disease stages—early, pre-symptom onset, post-symptom onset, and end stage.

## Methods

### Literature search

To obtain the initial primary article selection pool, PubMed searches were conducted in October 2014 to find all published articles with (“Amyotrophic Lateral Sclerosis” OR “ALS”) in the title or abstract AND (“transgenic mouse” OR “G93A”) in the title or abstract (Kim et al., 2015; Pfohl et al., 2015). Initial primary article selection pool exclusion criteria consisted of: non-English language articles; articles for which full-text pdf downloads were unavailable; and articles labeled as literature reviews. Articles were either downloaded using PubMed Central or from e-journal subscriptions available from the libraries of Georgia Institute of Technology and Emory University.

Information from the articles was entered systematically into a relational database using the software FileMaker Pro 13 Advanced (Filemaker, Inc.; Kim et al., 2015) and included stringent quality control procedures (Mitchell et al., 2015a). Aggregate data pertaining to cytokine levels of TG mice was mined using a predetermined list of cytokines (Table 1), which included alternate spellings [e.g., IL-1α, interleukin 1a, IL-1a, IL-alpha, IL-1(alpha), etc.]. Searches were performed in the article title, abstract, figure captions, and within figure text to find relevant articles from the primary article pool (Kim et al., 2015; Pfohl et al., 2015). These searches resulted in a study selection pool of 153 articles from which initial data was collected. The following list of inclusion and exclusion criteria was used to extract data, shown in Table 2, relevant to cytokine level.

**Table 1:** **Cytokine outline**.

**Cytokine**	**Type**	**Background description**
IL-1α	I	IL-1α and IL-1ß are the interleukins most directly associated with acute and chronic inflammation, and they share the same receptor and coreceptor IL-1RI and IL-1RacP, respectively (Dinarello, 2011).
IL-1β	I	
IL-12	I	IL-12 is known as a type I T cell stimulating factor, which can stimulate the growth and function of T cells, including tumor necrosis factor alpha (TNFα) (Hsieh et al., 1993). IL-12 P70 heterodimer promotes the differentiation of type I helper cells which produce more type I cytokines. IL-12 P40 homodimer is an antagonist that is generally present when levels of IL-12 P70 heterodimer are in excess (Suzuki et al., 2003).
TNF-α	I	TNFα is a type I cytokine released from M1 macrophages and is involved in triggering apoptosis (Kriegler et al., 1988).
GFAP	I	Glial fibrillary acidic protein (GFAP) has been shown to be upregulated in autoimmune diseases where TNFα and IL-1ßwere overexpressed (Von Boyen et al., 2004), causing glial scarring as the astrocytes interact with neural injuries (Ribotta et al., 2004).
IL-4	II	IL-4 is the interleukin primarily responsible for the differentiation of helper T cells to Th2 cells that participate in the anti-inflammatory response (Sokol et al., 2008).
IL-6	II	IL-6 was originally defined as a pro-inflammatory cytokine secreted by T cells and macrophages (Ferguson-Smith et al., 1988). However, IL-6 has more recently been shown to have anti-inflammatory properties when released by muscle cells (Pedersen, 2013).
IL-10	II	IL-10 is an anti-inflammatory cytokine commonly known as human cytokine synthesis inhibitory factor (CSIF) (Eskdale et al., 1997).

*These cytokines were included in this study due to their high prevalence in the primary article pool*.

**Table 2:** **Summary of data**.

**Cytokine**	**Total papers**	**Total ratios**	**References**
IL-4	6	7	Hensley et al., 2002, 2003; Kim et al., 2006; Beers et al., 2008; Chiu et al., 2008; Audet et al., 2012
IL-6	8	12	Hensley et al., 2002, 2003; Weydt et al., 2004; Xie et al., 2004; Kim et al., 2006; Chiu et al., 2008; Audet et al., 2012; Dibaj et al., 2012
IL-10	3	6	Hensley et al., 2002, 2003; Finkelstein et al., 2011
IL-1α	5	6	Hensley et al., 2003; Kiaei et al., 2006; Kim et al., 2006; Kassa et al., 2009; Neymotin et al., 2009
IL-1β	12	20	Hensley et al., 2003; Kang et al., 2003; Xie et al., 2004; Kiaei et al., 2006; Kim et al., 2006; Kassa et al., 2009; Neymotin et al., 2009; Fang et al., 2010; Zhao et al., 2010; Berger et al., 2011; Audet et al., 2012; Valente et al., 2012
IL-12	6	12	Hensley et al., 2002, 2003, 2006; Kiaei et al., 2006; Kim et al., 2006; Dibaj et al., 2012
TNF-α	18	39	Yoshihara et al., 2002; Hensley et al., 2003; Chen et al., 2004; Weydt et al., 2004; Xie et al., 2004; Perrin et al., 2005; Henkel et al., 2006; Kim et al., 2006; Zhang et al., 2008; Cheroni et al., 2009; Neymotin et al., 2009; Fang et al., 2010; Takeuchi et al., 2010; Finkelstein et al., 2011; Pollari et al., 2011; Audet et al., 2012; Valente et al., 2012
GFAP	46	164	Olsen et al., 2001; Yoshihara et al., 2002; Liu and Martin, 2006; Ohta et al., 2006; Pardo et al., 2006; Guan et al., 2007; Petrik et al., 2007; Yin et al., 2007; Beers et al., 2008; Pitzer et al., 2008; Poesen et al., 2008; Shibata et al., 2008; Boucherie et al., 2009; Cheroni et al., 2009; Kassa et al., 2009; Keller et al., 2009, 2011; Ringer et al., 2009; Sekiya et al., 2009; Yang et al., 2009, 2011; Guo et al., 2010, 2011; Israelsson et al., 2010; Jokic et al., 2010; Moreno-Igoa et al., 2010; Shimazawa et al., 2010; Steinacker et al., 2010; Suzuki et al., 2010; Tsai et al., 2010; Berger et al., 2011; Finkelstein et al., 2011; Genestine et al., 2011; Zhu and Sheng, 2011; Gifondorwa et al., 2012; Miquel et al., 2012; Valente et al., 2012
Type I total	56	241	Olsen et al., 2001; Hensley et al., 2002, 2003, 2006; Yoshihara et al., 2002; Kang et al., 2003; Chen et al., 2004; Weydt et al., 2004; Xie et al., 2004; Perrin et al., 2005; Henkel et al., 2006; Kiaei et al., 2006; Kim et al., 2006; Liu and Martin, 2006; Ohta et al., 2006; Pardo et al., 2006; Guan et al., 2007; Petrik et al., 2007; Yin et al., 2007; Beers et al., 2008; Pitzer et al., 2008; Poesen et al., 2008; Shibata et al., 2008; Zhang et al., 2008; Boucherie et al., 2009; Cheroni et al., 2009; Kassa et al., 2009; Keller et al., 2009, 2011; Neymotin et al., 2009; Ringer et al., 2009; Sekiya et al., 2009; Yang et al., 2009, 2011; Fang et al., 2010; Guo et al., 2010, 2011; Israelsson et al., 2010; Jokic et al., 2010; Moreno-Igoa et al., 2010; Shimazawa et al., 2010; Steinacker et al., 2010; Suzuki et al., 2010; Takeuchi et al., 2010; Tsai et al., 2010; Zhao et al., 2010; Berger et al., 2011; Finkelstein et al., 2011; Genestine et al., 2011; Pollari et al., 2011; Audet et al., 2012; Dibaj et al., 2012; Gifondorwa et al., 2012; Miquel et al., 2012; Valente et al., 2012
Type II total	10	25	Hensley et al., 2002, 2003; Weydt et al., 2004; Xie et al., 2004; Kim et al., 2006; Beers et al., 2008; Chiu et al., 2008; Finkelstein et al., 2011; Audet et al., 2012; Dibaj et al., 2012

*Information gathered on each cytokine that was suitable for use in the statistical analysis*.

### Study inclusion criteria

- Cytokines and small proteins that were directly related with cytokine effects. Table 1 provides a brief outline of the cytokines measured in this study (Ferguson-Smith et al., 1988; Kriegler et al., 1988; Hsieh et al., 1993; Eskdale et al., 1997; Suzuki et al., 2003; Ribotta et al., 2004; Von Boyen et al., 2004; Sokol et al., 2008; Dinarello, 2011; Pedersen, 2013). Although glial fibrillary acid protein (GFAP) is not a cytokine, it was categorized with type I cytokines, since GFAP levels correlates directly with an increase in the activity of pro-inflammatory cytokines, and GFAP increases with damage to the central nervous system (Ribotta et al., 2004)
- Measures dealing with densities, mRNA or protein levels, and fold change for each of the cytokines
- High transgene copy SOD1 G93A murine model (TG) B6SJL-Tg.

### Study exclusion criteria

- Data that did not measure cytokine counts or expressions either directly or indirectly
- Measures with treatment on the mouse model
- Data in which there was no evident manner to normalize the results
- Purely *in vitro* data
- Cytokine levels measured greater than 136 days (see Section Data Normalization).

### Data normalization

In order to account for the variation of cytokine level measurement methods between the many papers, cytokine level data were normalized by the calculation of ratios of TG to WT cytokine levels. In the case that a TG value did not have a WT value for corresponding time point, if at least one WT timepoint was present, that one WT value was used to normalize the TG value as the WT value was observed to be constant with age (Ringer et al., 2009). Each ratio was then weighted according to the sample size found for its respective study. When sample size values were not explicitly or clearly reported in papers (Kang et al., 2003; Weydt et al., 2004; Xie et al., 2004; Kiaei et al., 2006; Kassa et al., 2009; Finkelstein et al., 2011; Audet et al., 2012; Dibaj et al., 2012), a value of 1 was used for the sample size in the statistical analysis in order to allow for the data to make a conservative, yet deserved, impact on the analysis.

### Disease stages

The disease stages were determined by taking the mean and standard deviation of all reported time of onset (97 ± 20), and of all reported time of death (135 ± 19) in the papers included, and marking one standard deviation before and after these means as critical points for the ranges. The resultant four defined time periods are as follows: 0–76 days (early stage), 77–96 days (pre-onset stage), 97–116 day (post-onset stage), 117–135 days (end stage). Data after the mean time of death (135 days) was excluded, since data was only available for two type I cytokines, TNF-α or GFAP.

### Analysis

A right tailed *t*-test was used to test the statistical significance of the means of each cytokine level against the null hypothesis that the cytokine levels between TG and WT was unchanged for each time period or disease stage. Shapiro-Wilk test was used to determine that the TG/WT ratio data was normally distributed. To insure a conservative assessment, Bonferroni correction was used to lower the defined statistical significance to *p* < 0.002. One-way ANOVA was used to test the significance of means for each defined time period. ANCOVA was used to test the significance of the rate of change of cytokine levels among the different cytokines, and among grouped type I and type II cytokines, for each defined time period; the significance level for this comparison was set as α = 0.05. MATLAB (The Mathworks, Inc.) was used to perform the statistical analysis. A meta-analysis with fixed effects was also conducted using *metan* in Stata (Statacorp, 2015). Since meta-analysis is more conservative in terms of data inclusion criteria, meta-analysis for the studies with type I cytokines was done. While the forest plots look at studies compared to each other, the meta-analysis using normalized data operates under a similar fixed effects model in which the value of each study, instead of the study itself, is weighted using sample size.

## Results

**Figure 1:**
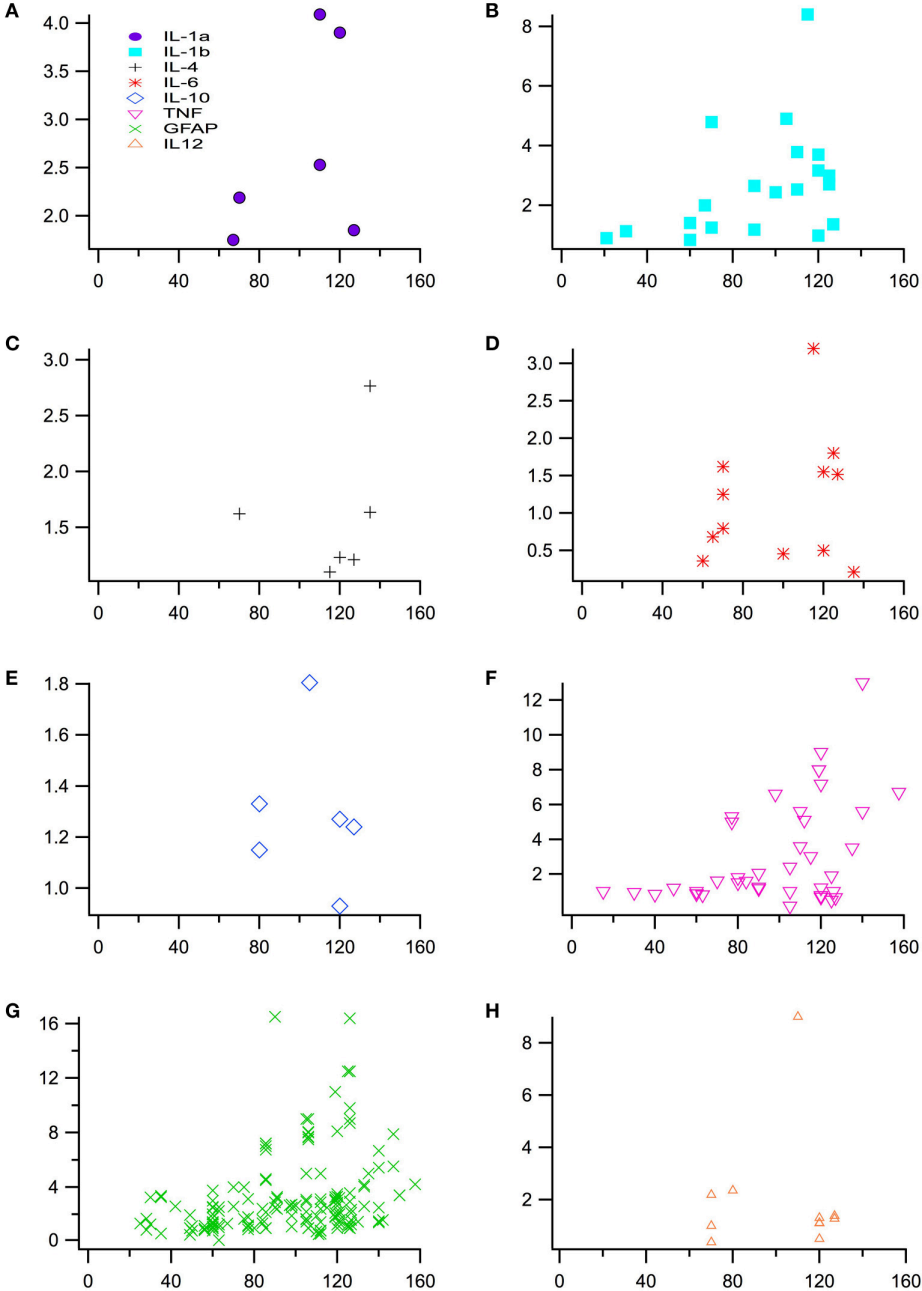
**Normalized cytokine levels used to assess differences between SOD1 G93A transgenic mice (TG) and wild type (WT) mice over time or post-natal age (days)**. Plot of individual cytokine levels, **(A)** IL-1α, **(B)** IL-1ß, **(C)** IL-4, **(D)** IL-6, **(E)** IL-10, **(F)** TNF-α **(G)** GFAP, and **(H)** IL-12, used in the analysis.

**Figure 2:**
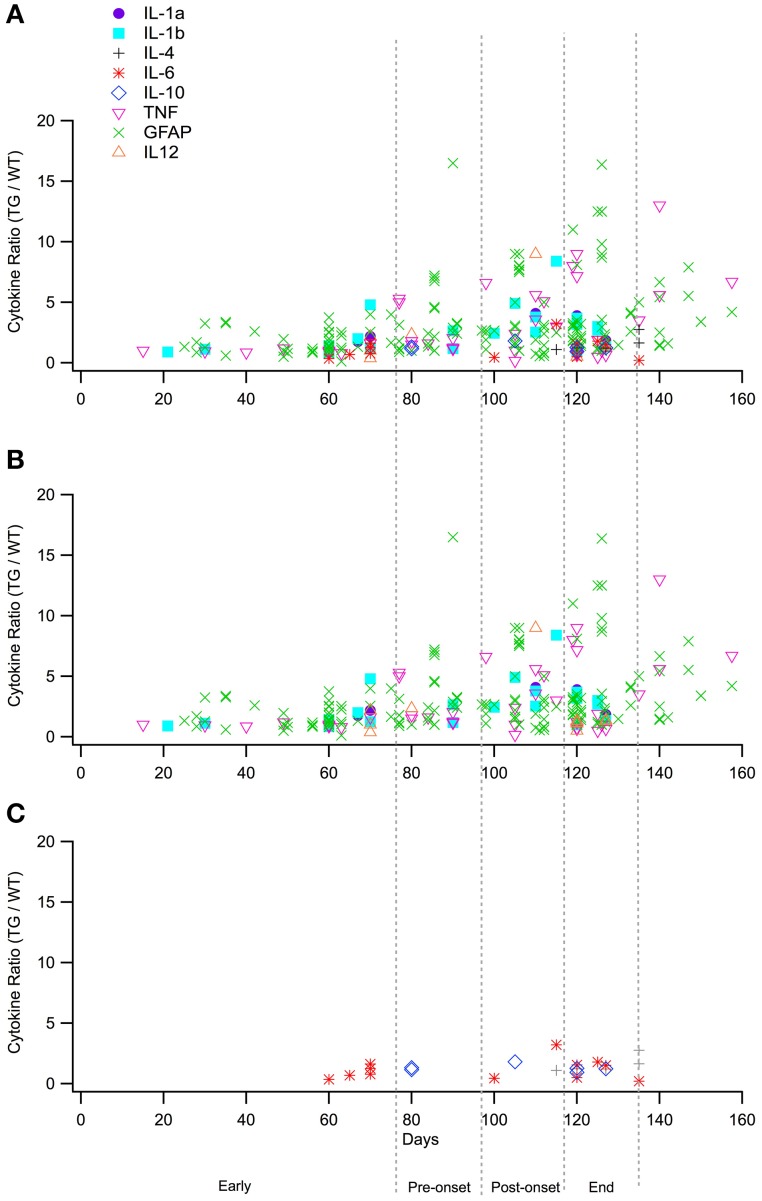
**Normalized cytokine levels over time separated by the four stages utilized for statistical analysis, early (0–76 days), pre-onset (77–96 days), post-onset (97–116 days), and end stages (117–135 stages)**. **(A)**. Plot of all raw data cytokine ratios (TG/WT) for type I and type II cytokines. **(B)**. Cytokines ratios (TG/WT) for type I cytokines only. **(C)**. Cytokine ratios (TG/WT) for type II cytokines only.

Experimental *in vivo* data on cytokines levels in transgenic (TG) SOD1 G93A ALS mice and wild type (WT) mice was utilized from a total of 66 peer-reviewed articles, which met the study inclusion criteria. The ratio of TG to WT cytokine levels at each examined time point was calculated, resulting in total of 266 TG/WT ratios. TG/WT cytokine level ratio is plotted for each specific cytokine type over the TG mouse life span (Figure 1). Figure 2A illustrates the data included in our meta-analysis, separated by disease stage. Individual cytokines are aggregated and presented by their corresponding type, type I (Figure 2B) or II (Figure 2C). Since pro-inflammatory type I cytokines are generally viewed as having a larger role in the progression of ALS, type II (IL-4, IL-6, and IL-12) cytokines have been less studied in the experimental literature; consequently, there is less type II cytokine data.

### Differences in TG levels compared to WT levels in and across each stage

**Figure 3:**
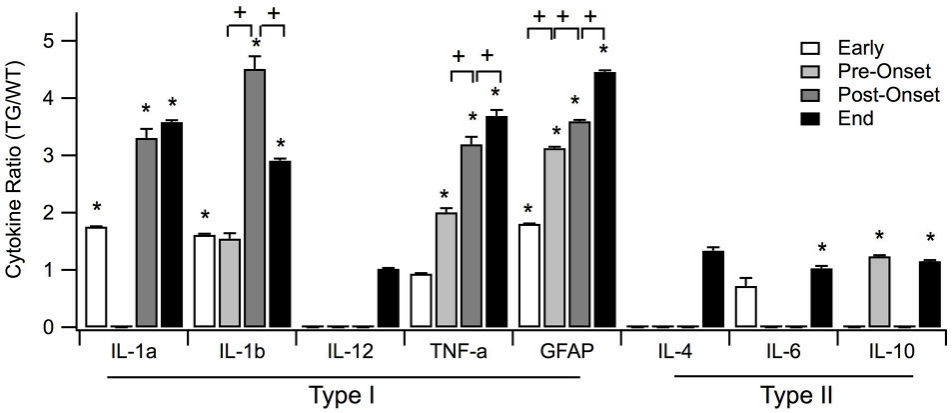
**Means of normalized cytokine levels (TG/WT) for each of the four SOD1 G93A ALS mouse disease stages: early, pre-onset, post-onset, and end**. Statistically significant differences between SOD1 G93A transgenic mice (TG) and wild type (WT) levels within a disease stage are marked with asterisk (^*^). Statistically significant positive or negative fold changes across consecutive disease stages are represented by plus (+) or minus (−), respectfully. Significance was adjusted to *p* < 0.002 using Bonferroni correction. Error bars represent confidence interval.

In Figure 3, the means of the fold change for all cytokines within each time period is positive with the exception of the means of IL-6 and TNF-α. At the early stage, the fold changes of IL-6 (0.7214) and TNF-α (0.9369) appear to be qualitatively less than WT, but are not statistically significant. The fold changes of all the other cytokines are greater than one, indicating that the TG cytokine levels were overexpressed relative to WT cytokine levels.

**Figure 4:**
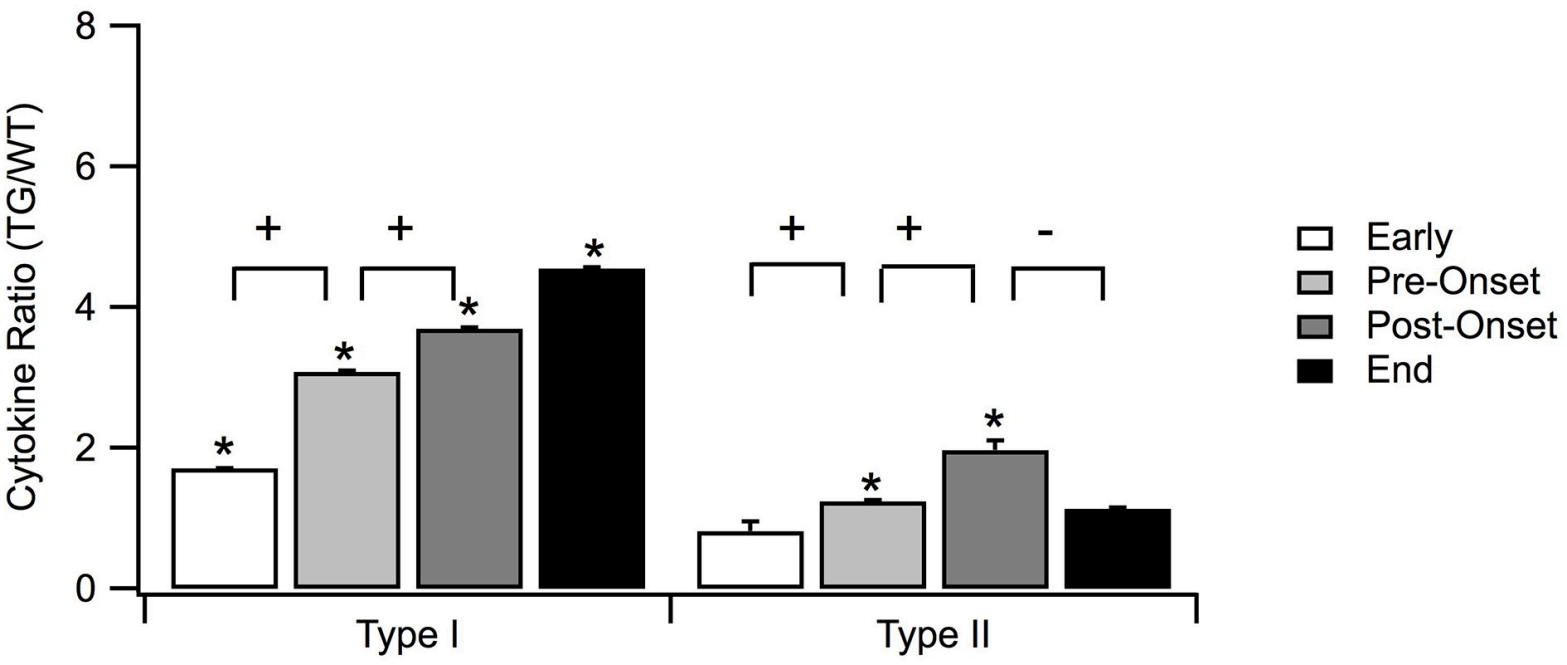
**Means of normalized type I and type II for each of the four SOD1 G93A ALS mouse disease stages: early, pre-onset, post-onset, and end stage**. Type I cytokines were significantly higher than type II cytokines for each of the four stages. Statistically significant differences between SOD1 G93A transgenic mice (TG) and wild type (WT) levels within a disease stage are marked with asterisk (^*^). Statistically significant positive or negative fold changes across consecutive stages are represented by plus (+) or minus (–), respectfully. Significance was adjusted to *p* < 0.006 using Bonferroni correction. Error bars represent confidence intervals.

Among the individual type I cytokines, IL-1α, IL-1ß, and GFAP show significance in their fold increase (*p* < 0.0001) across all of the four stages. TNF-α shows significance in fold increase in all stages except for pre-onset, and IL-12 does not show any significance at stages where sufficient data was present to be tested. Across aggregated type I cytokines, increase of TG levels relative to WT levels is statistically significant for each disease stage (Figure 4).

Of type II cytokines, IL-6, and IL-10 show significance in increase in all stages where sufficient data were present (Figure 3, marked with ^*^). In contrast, IL-4 showed statistically insignificant increases in both the early and end disease stages (Figure 3).

Across the four stages for type I cytokines, the GFAP level expression increased significantly across all stages (Figure 3, marked with +). TNF-α expression increased significantly across the first three stages. IL-β levels are increased in the early, post-onset and end stages, but not for pre-onset. IL-1α, IL-12, IL-4, IL-6, and IL-10 contain missing data in crucial stages to reach conclusive results. Overall, the rate of type I cytokine levels increased significantly across the first three stages (Figure 4, marked with +). Type II cytokines expression increased significantly across the first three stages (Figure 4, marked with +), but decreased significantly from the post-onset stage to the end stage (Figure 4, marked with –).

### Fold change difference between type I and type II cytokines

In Figure 4, the mean fold increase of type I is not only greater than that of type II across all four stages, but the difference was also statistically significant in all four stages. The difference between the means increases from early to pre-onset stage. At the post-onset stage, the difference does not continue this increase, but, instead, decreases slightly as a result of the increase in anti-inflammatory response to mediate the overexpression of the inflammatory response. Toward the end stage, the inability of the pathological system to compensate for the increase in pro-inflammatory response to maintain dynamic stability is seen through the abrupt increase in the difference of the fold changes.

### Meta-analysis of type I cytokines

Due to the type of data and the experimental heterogeneity of the assessed measures and each study's precision, standard meta-analysis as a stand-alone method does not provide sufficient dynamical assessment (Higgins et al., 2003), in terms of examining both cytokine rate changes and fold changes. Nonetheless, it is valuable for assessing the contributions of individual studies and providing very conservative affirmation of the quantified significance of fold change in cytokines. From the meta-analysis, there is no statistical significance in the results between each of the studies in the early and post-onset stages, but there is statistical significance in that of pre-onset and end stages as shown in the results from the forest plot (see Supplementary Table 1). Therefore, the conservative results of the meta-analysis are supportive of the results described above using traditional statistics with Bonferroni correction. Finally, the I^2^ statistics for each of these stages with the exception of pre-onset stage indicate that, as would be expected, there is considerable heterogeneity between studies.

### Differences in rate of change of fold changes between individual cytokines, and between type I and type II cytokines

In Table 3, for each of the four time periods, both the rate of change of each cytokine compared to the average rate of change, and the rate of change of each type compared to the average rate of change in each stage were not significant.

**Table 3:** **Rate of change of type I vs. type II cytokine levels at each stage**.

**Cytokine**	**Early stage**		**Pre-onset stage**		**Post-onset stage**		**End stage**	
	**Slope**	***p*-value**	**Slope**	***p*-value**	**Slope**	***p*-value**	**Slope**	***p*-value**
IL-4	0.0525	–	–	–	0.114	–	0.048	0.7967
IL-6	0.086	0.678	–	–	0.183	0.8791	0.079	0.7575
IL-10	–	–	−0.022	–	0.114	–	0.055	0.8923
IL-1α	0.1467	0.694	–	–	0.114	–	−0.293	0.4438
IL-1ß	0.023	0.6311	−0.022	–	0.322	0.4816	−0.193	0.5885
IL-12	0.0525	–	−0.022	–	0.114	–	0.060	0.7262
TNF-α	−0.001	0.3854	−0.193	0	−0.018	0.6047	−0.197	0.4126
GFAP	0.0079	0.4669	0.149	0	−0.031	0.4595	0.229	0.0404
Average	0.0525	0.3918	−0.022	0.4771	0.114	0.5279	−0.034	0.759
Type I	0.0104	0.1837	0.0658	–	−0.0227	0.8224	0.242	0.356
Type II	0.0964	0.1837	–	–	0.0655	0.8224	0.05	0.356
Average	0.0534	0.0991	–	–	0.0214	0.9134	0.146	0.1619

*The p-value for the average slope shows the significance of slope differing from zero. None of the rates of change of cytokines or types of cytokines are significantly different from their respective average slopes*.

## Discussion

While end stage cytokine levels have been described in various studies on the TG model, our analysis especially sheds light on the previously underrepresented early stage and pre-onset cytokine levels (McCombe and Henderson, 2011). Overexpression of individual cytokines was expected near end stage. However, interestingly, overexpression of individual cytokines also occurred in the early and pre-onset stages, well before the measurable appearance of physical symptoms. GFAP, a clear indicator of inflammation, is expressed twice the WT cytokine levels in the early stage, well before the onset of the disease, and the protein continues to significantly increase throughout disease progression, reaching almost 4.5 times the WT counterpart (Siemionow et al., 2009).

In addition to the expected increase in pro-inflammatory type I cytokine levels in stages after onset, type I cytokine levels in TG mice also increased relative to WT mice significantly before the onset of the disease. This early deviation from the normal aggregate type I cytokine levels reveals the start of the inflammatory response well before the symptomatic stage. Overall, as a group, type II cytokines show significantly different means only in the pre-onset and post-onset stages (Figure 4, marked with ^*^). Unfortunately, there is a general lack of data to draw strong conclusions regarding expression of individual type II cytokines.

Aggregating the individual cytokines respective to their types yields a more holistic view of the pro- and anti-inflammatory balance in ALS. Type II cytokine level rates increase appropriately across the first three stages to counteract the overexpression of type I cytokines. However, at the end stage, type II cytokines fail to maintain this appropriate response, thereby facilitating the increased effect of pro-inflammatory response on neuronal damage. This disruption in immunoregulatory balance is a remnant of the overall regulatory instability that is a contributor to ALS (Irvin et al., 2015).

In response to the disruption in the balance between pro and anti-inflammatory responses via the increase in type I cytokine levels, type II cytokine levels should also increase to mediate this imbalance (Philips and Robberecht, 2011). However, type II exhibits the opposite of the appropriate response. Although both types of cytokines increase significantly over the first three stages of the disease, type I cytokine levels plateau at the end stage, but type II cytokine levels decrease significantly (Figure 4). The insignificant change in type I cytokine levels in the end stage may have an impact on the decrease in expression of type II cytokine levels. Interestingly, after onset, the immune system appears to balance the rates of change of the cytokines as the disease progresses, although the mean cytokine levels remain elevated.

Despite the pathological state, which causes TG mice cytokine levels to be generally overexpressed, the rates at which the cytokines change across time for each stage do not indicate an aberrant cytokine. Rather, the increase or decrease of each cytokines aligns with the average change of cytokine levels in the system. This could indicate that the mechanisms which causes changes of cytokine levels over the disease progression occurs as a result of a system response against inflammation caused by the ALS pathology, such as gliosis, and not as a direct cause of the ALS pathology, itself. This is similar to recent hypotheses suggestion that the hallmark amyloid beta plaques in Alzheimer's disease could be a side effect rather than the direct cause of cognitive decline (Foley et al., 2015). The lower prevalence of antecedent disease in clinical ALS population (Mitchell et al., 2015b) indicates that inflammation is likely a side effect, rather than a precursor, of ALS.

The results of our study suggest that inflammation may have more of a crucial role in the earlier stages of ALS. Although gliosis through the perturbation of microglia and astrocytes by cytokine molecules act as protective barriers for normal injuries, unregulated, continued increase of pro-inflammatory cytokine levels in a pathological state can cause unnecessary gliosis that cause damage and is harmful (Papadimitriou et al., 2010). As a result, immunoregulatory treatments to decrease inflammation by increasing the anti-inflammatory response may be more effective when administered earlier in the course of ALS. Such treatments administered later in the disease may have little, if any, impact since the pro-inflammatory cytokines have already reached the crucial levels. Interestingly, similar observations have been made in regards to timing the treatment of neuroinflammation in secondary spinal cord injury (Mitchell and Lee, 2008).

The traditional school of thought has been to utilize type I cytokine inhibitors or type II cytokine activators to limit the high levels of inflammation. For example, a historical study that used COX-2 inhibitors as treatments showed significant decrease in inflammatory responses (McGeer and McGeer, 2002). However, the results of the present study reveal that the ability to actually modulate the immunoregulatory response, and do so with precise timing, may be more favorable. In fact, recent studies attempting immunoregulatory modulation have shown promise. For example, immunizations with a myelin-derived antigen have been utilized to stimulate immunoregulatory cell recruitment, ultimately attenuating disease progression in the ALS mouse model (Kunis et al., 2015). Other strategies have included immunomodulatory effects of human mesenchymal stem cells on peripheral blood mononuclear cells in ALS patients (Kwon et al., 2014), altering the native environment of neuroprotective T-cells (Mesnard-Hoaglin et al., 2014), and the usage of IgG antibodies as biomarkers (Schwartz and Baruch, 2014).

Perhaps the most exciting therapeutic hypothesis to date is to boost native autoimmunity. Autoimmune T-cells are part of a cellular network which, to operate efficiently and safely, requires tight regulation by other immune cell populations, such as regulatory T cells, which are indispensable for maintenance of immunological self-tolerance and homeostasis (Schwartz and Baruch, 2014). It has been previously suggested that dysregulation of the balance between peripheral immune suppression, on one hand, and protective autoimmunity, on the other, is an underlying mechanism in the emergence and progression of the neuroinflammatory response associated with chronic neurodegenerative diseases and brain aging (Schwartz and Baruch, 2014). The dynamical instability between type I and II cytokines in SOD1 G93A ALS mice, as identified in the present study, is supportive of the immune dysregulation hypothesis. The ability to exploit the intricate relationships and immunoregulatory pathways via cellular therapy is an exciting path for the future of ALS treatment (Rizzo et al., 2014).

Finally, the testing of anti-inflammatory treatments in various time ranges, and not simple presence or absence of anti-inflammatory treatment, may achieve not only a deeper understanding of the possible mechanisms of the balance between pro- and anti-inflammatory responses, but also give rise to a more effective treatment that may further extend life expectancy.

## Conclusions

It is evident that the levels of most of the individual cytokines in TG are generally higher than their corresponding WT counterpart for all four disease stages. Pro-inflammatory type I cytokines are expressed at higher levels than anti-inflammatory type II cytokines across all stages. Moreover, type I cytokine levels increase significantly across the first three stages, whereas type II cytokine levels increase significantly until the post-onset stage and then decrease at the end stage. The pronounced increase in difference between inflammatory and anti-inflammatory throughout the life span of ALS mice is likely due to the inability of the pathological system to maintain stability (Mitchell and Lee, 2012). In fact, type I normalized TG levels are ~four times greater than WT in the post-onset and end stages, whereas the type II normalized TG levels are only two times greater for the post-onset stage and almost equivalent to WT levels at end stage. Early intervention and immunoregulatory modulation addressing the dynamic neuroinflammatory instability are two keys to future treatment success. Finally, assessment of homeostatic regulation failure in inflammation and other cellular pathways remains a promising avenue for identifying the underlying etiology of ALS (Irvin et al., 2015).

## Author contributions

AJ processed data; co-designed and performed statistical analysis; aided in data interpretation; and drafted initial manuscript. BM processed data; co-designed and performed statistical analysis; aided in data interpretation. EM processed data; aided in data formatting and analysis; aided in the initial manuscript draft. CM conceived the study; participated in its design, coordination, and interpretation; and drafted the final manuscript. All authors read and approved the final manuscript.

## Funding

Funding provided by USA National Institute of Health grants NS081426 and NS069616 to CM.

### Conflict of interest statement

The authors declare that the research was conducted in the absence of any commercial or financial relationships that could be construed as a potential conflict of interest.

## Abbreviations

ALS: Amyotrophic Lateral Sclerosis
SOD1: superoxide dismutase gene
TG: transgenic
GFAP: Glial fibrillary acidic protein
TNF-α: Tumor necrosis factor alpha
IL: Interleukin.
