# Supplementary material for: Type I Vs. Type II Cytokine Levels as a Function of SOD1 G93A Mouse Amyotrophic Lateral Sclerosis Disease Progression
Source: Front Cell Neurosci. 2015 Dec 1;9:462. doi: 10.3389/fncel.2015.00462 (PMC4664727; doi:10.3389/fncel.2015.00462)
Supplement: Supplementary file 1 [file Table1.PDF]

Supplementary Table 1A. Meta-analysis of type I cytokines for early stage. $I^2 = 70.0$ %; $p < 0.05$.

Study	Mean (95% C.I.)	Weight (%)
Olsen et al. (2001)	1.37 (0.84, 1.89)	2.5
Yoshihara et al. (2002)	1.10 (0.82, 1.38)	8.97
Xie et al. (2004)	0.93 (0.72, 1.13)	15.95
Kiaei et al. (2006)	0.91 (0.80, 1.02)	57.21
Liu and Martin (2006)	1.91 (-0.88, 4.71)	0.09
Cheroni et al. (2009)	0.68 (-0.90, 2.26)	0.28
Keller et al. (2009)	1.69 (1.03, 2.35)	1.58
Sekiya et al. (2009)	1.92 (-0.17, 4.01)	0.16
Neymotin et al. (2009)	1.05 (0.36, 1.74)	1.44
Steinacker et al. (2010)	0.95 (0.70, 1.20)	10.99
Guo et al. (2010)	1.94 (0.12, 3.76)	0.21
Yang et al. (2011)	3.63 (2.59, 4.66)	0.64
Overall	0.98 (0.90, 1.06)	100

Supplementary Table 1B. Meta-analysis of type I cytokines for pre-onset stage. $I^2 = 33.8$ %; $p < 0.05$.

Study	Mean (95% C.I.)	Weight (%)
Yoshihara et al. (2002)	3.47 (-0.32, 7.27)	0.05
Cheroni et al. (2009)	1.48 (1.13, 1.82)	0.56
Keller et al. (2009)	2.25 (0.39, 4.11)	0.23
Fang et al. (2010)	1.20 (1.11, 1.29)	93.13
Yang et al. (2011)	1.22 (1.13, 1.31)	0.03
Overall	1.22 (1.13 1.31)	100

Supplementary Table 1C. Meta-analysis of type I cytokines for post-onset stage. $I^2 = 93.9$ %; $p < 0.05$.

Study	Mean (95% C.I.)	Weight (%)
Yoshihara et al. (2002)	4.55 (-1.13, 10.23)	0.01
Xie et al. (2004)	3.65 (0.19, 7.11)	0.02
Kiaei et al. (2006)	4.69 (-2.63, 12.01)	0
Liu and Martin (2006)	0.82 (0.77, 0.86)	96.26
Cheroni et al. (2009)	3.75 (0.01, 7.49)	0.01
Keller et al. (2009)	2.41 (0.99, 3.82)	0.1
Sekiya et al. (2009)	3.95 (1.04, 6.36)	0.02
Neymotin et al. (2009)	3.82 (3.32, 4.32)	0.81
Steinacker et al. (2010)	1.57 (1.29, 1.84)	2.67
Guo et al. (2010)	1.33 (-0.27, 2.92)	0.08
Yang et al. (2011)	13.10 (-20.03, 46.22)	0

Audet et al. (2012)	5.70 (-1.78, 13.18)	0
Overall	0.87 (0.82, 0.91)	100

Supplementary Table 1D. Meta-analysis of type I cytokines for end stage. $I^2 = 84.5$ %; $p < 0.05$.

**Study**	**Mean (95% C.I.)**	**Weight (%)**
Olsen et al. (2001)	3.00 (2.50, 3.51)	0.06
Yoshihara et al. (2002)	5.53 (-0.08, 11.13)	0
Hensley et al. (2003)	2.28 (-2.47, 7.04)	0
Xie et al. (2004)	2.30 (1.19, 3.41)	0.01
Ohta et al. (2006)	2.08 (0.82, 3.33)	0.01
Hensley et al. (2006)	1.11 (1.09, 1.12)	99.72
Petrik et al. (2007)	1.83 (0.85, 2.81)	0.02
Cheroni et al. (2009)	5.45 (1.99, 8.91)	0
Kassa et al. (2009)	2.87 (-0.31, 6.04)	0
Keller et al. (2009)	4.17 (0.52, 7.83)	0
Guo et al. (2010)	1.95 (0.43, 3.47)	0.01
Fang et al. (2010)	0.81 (0.50, 1.12)	0.16
Yang et al. (2011)	15.70 (-20.19, 51.58)	0
Finkelstein et al. (2011)	4.25 (2.17, 6.33)	0
Valente et al. (2012)	1.46 (-0.66, 3.57)	0
Overall	1.11 (1.10, 1.12)	100
